# Seasonal dynamics of *Amblyomma sculptum*: a review

**DOI:** 10.1186/s13071-022-05311-w

**Published:** 2022-06-06

**Authors:** Luiza Gabriella Ferreira de Paula, Rafael Moreira do Nascimento, Artur de Oliveira Franco, Matias Pablo Juan Szabó, Marcelo Bahia Labruna, Caio Monteiro, Felipe da Silva Krawczak

**Affiliations:** 1 Programa de Pós-Graduação em Ciência Animal, Universidade Federal de Goiás (UFG), Avenida Esperança, s/n, Campus Samambaia, Goiânia, Goiás 74690-900 Brazil; 2 Programa de Pós-Graduação em Ciências Veterinárias, Universidade Federal Rural do Rio de Janeiro, Seropédica, Rio de Janeiro 23890-000 Brazil; 3 Instituto de Patologia Tropical e Saúde Pública, Universidade Federal de Goiás, Goiânia, Goiás 74.605-050 Brazil; 4 Laboratório de Ixodologia, Faculdade de Medicina Veterinária, Universidade Federal de Uberlândia, Uberlândia, MG Brazil; 5 Departamento de Medicina Veterinária Preventiva e Saúde Animal, Faculdade de Medicina Veterinária e Zootecnia, Universidade de São Paulo, São Paulo, SP Brazil; 6 Escola de Veterinária e Zootecnia, Universidade Federal de Goiás, Avenida Esperança, s/n, Campus Samambaia, Goiânia, Goiás 74.690-900 Brazil

**Keywords:** Ecology, Behavioral diapause, Hosts, Tick collection

## Abstract

**Background:**

*Amblyomma sculptum* is a hard tick that is associated with domestic animals and the transmission of Brazilian spotted fever. This association has motivated several field studies on this ixodid tick within its distribution area in South America. Thorough knowledge of the seasonal dynamics of *A. sculptum* in different ecological scenarios is required in order to better understand the biological characteristics of this tick and develop techniques for the control and prevention of diseases transmitted by this vector. In this article, we systematically review the seasonal dynamics of *A. sculptum* and tick collection methodology.

**Methods:**

A systematic search of the Scopus, Web of Science, PubMed and Scielo databases was carried out for articles (including dissertations and theses) on the population dynamics of *A. sculptum*. The inclusion criterion was the report of seasonal dynamic studies on *A. sculptum* through surveys carried out for at least 1 year with, as methodology, tick collection in the environment and/or tick count/collection on *A. sculptum* primary hosts (horses or capybaras). Studies carried out before the reclassification of *Amblyomma cajennense* sensu lato in 2014, which referred to *Amblyomma cajennense* in areas where it is currently known that only *A. sculptum* occurs, were also included. Articles meeting the inclusion criterion, but not available in online databases, were also added based on the authors’ experience on the subject. Sixteen articles and one thesis were selected for inclusion in this systematic review.

**Results:**

Most of the studies were carried out in the southeastern region of Brazil, with a few also carried out in the northeast, center-west and south of Brazil and northwest of Argentina. Five techniques/methods were applied across these studies: CO_2_ traps, dragging, flagging, visual searches and tick counting on animals, used alone or in combination.

**Conclusion:**

Seasonal dynamics of *A. sculptum* was found to be similar in almost all of the areas studied, with larvae predominating during the autumn, nymphs in the winter and adults in the spring and summer.

**Graphical abstract:**

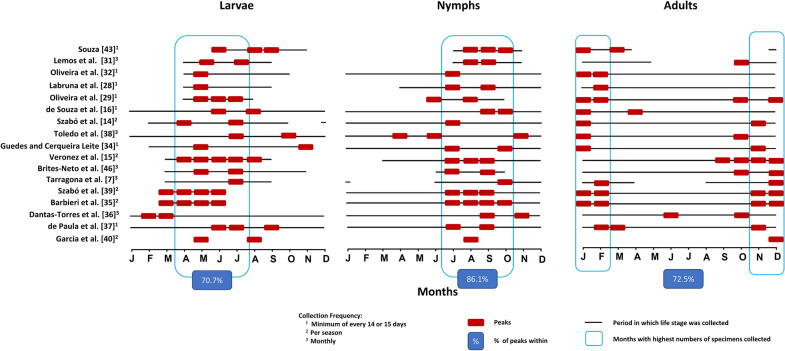

**Supplementary Information:**

The online version contains supplementary material available at 10.1186/s13071-022-05311-w.

## Background

*Amblyomma sculptum* Berlese, 1888, is a hard tick belonging to the *Amblyomma cajennense* species complex, alongside *Amblyomma cajennense* sensu stricto (Fabricius, 1787), *Amblyomma interandinum* Beati, Nava and Cáceres, 2014, *Amblyomma mixtum* Koch, 1844, *Amblyomma patinoi* Labruna, Nava and Beati, 2014 and *Amblyomma tonelliae* Nava, Beati and Labruna, 2014 [1]. Although capybaras, horses and tapirs are the preferred hosts [2], all stages of *A. sculptum* are considered to be aggressive towards humans [3, 4] and have been associated with the transmission of *Rickettsia rickettsii,* the agent of Brazilian spotted fever (BSF), a major tick-borne zoonosis in Brazil [5, 6]. *Amblyomma sculptum* is distributed over large areas in South America, currently encompassing the entire Cerrado and Pantanal biomes and a large part of the devastated Atlantic forest biome in Brazil [1, 2, 7], and may play a role in the transmission of other pathogens, such as *Theileria equi* to horses [8]*.*

Encounters between hosts and ticks, and consequently the transmission of pathogens, is influenced by tick behaviors [9, 10]. Therefore, basic knowledge of the ecological preferences and behaviors of ticks is indispensable for understanding the epidemiology of tick-borne diseases and for elaborating and implementing effective strategic control programs against *A. sculptum* in animals and in the environment.

In the context of the numerous studies that have been conducted on the seasonal dynamics of *A. sculptum*, the aim of the present study was to assemble all accessible information published on this topic.

## Methods

A systematic review of articles on the population dynamics of *A. sculptum* was conducted, with the Scopus, Web of Science, PubMed and Scielo databases searched for relevant articles (including dissertations and theses). These databases were chosen because they are among the largest and most prominent databases for indexing abstracts of scientific articles and because of the quality of the journals indexed on these platforms. The search of these databases was performed using the following keywords: tick species name (*Amblyomma cajennense* or *Amblyomma sculptum*) AND “seasonal dynamics”; articles in Spanish, English and Portuguese were considered.

The inclusion criterion was articles that reported on seasonal dynamic studies on *A. sculptum* through surveys carried out for at least 1 year and that had as methodology tick collection in the environment and/or tick count/collection on *A. sculptum* primary hosts (*Equus caballus* [horse] and *Hydrochoerus hydrochaeris* [capybaras]). Studies carried out before the reclassification of *Amblyomma cajennense* sensu lato in 2014, which referred to *A. cajennense* in areas where it is currently known that only *A. sculptum* occurs [1, 2], were also included in the review. Articles that met the inclusion criterion, but which were not found through searching the databases, but which the authors knew due to experience in the subject, were also added. Ultimately, 16 articles and one thesis were selected for inclusion in this systematic review. Although the PRISMA flowchart was broadly followed, it is not reported in the article due to the relatively low number of articles that fulfilled the inclusion criterion and the careful hand-picking of the relevant articles, including those from outside the on-line databases, which represented a slight divergence from the PRISMA flow chart.

Data from each article were compiled into an Excel® spreadsheet (Microsoft Corp., Redmond, WA, USA), in which the following parameters were recorded: authors; year(s) of tick collections; location; state/country and coordinates; biome; duration and frequency of collections; methods used for tick capture; stages collected; months during which each stage was collected; months in which peaks occurred for each stage; animals found at and near each location where collections took place; and whether the study site had any confirmed cases of BSF or any tick-borne rickettsia until the time the study was concluded.

Maps showing *A. sculptum* distribution and the locations of the seasonal dynamic studies were produced using the QGIS software [11]. These maps were constructed after the data on all the seasonal dynamic study location coordinates and the location coordinates of studies citing *A. sculptum* distribution had been compiled into the Microsoft Excel® spreadsheet. An image illustrating the ticks’ life-cycle was drawn up using Adobe Illustrator [12]. Information on the mean photoperiod [13] of every month in which it was reported that each stage was collected in higher quantities was also compiled into a Microsoft Excel® spreadsheet.

## Results and discussion

### *Amblyomma sculptum* distribution

**Fig. 1:**
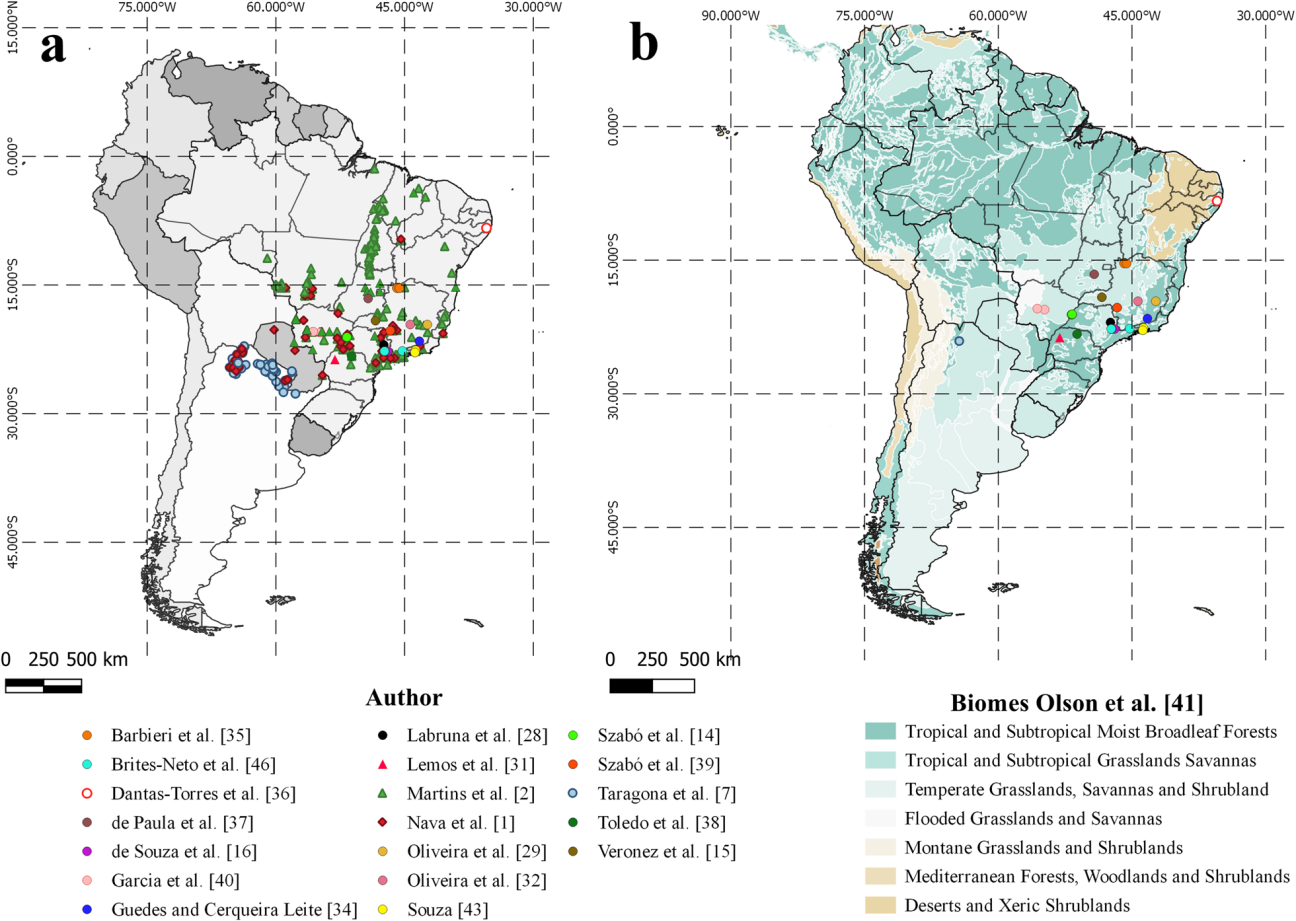
Currently known distribution of *Amblyomma sculptum* ticks (**a**) and locations of seasonal dynamic studies conducted between 1990 and 2022, with emphasis on the types of biomes for each location (**b**)

The occurrence of *A. sculptum* has been confirmed in Bolivia, Brazil, Paraguay and Argentina (Fig. 1a) [1]. This tick species is mainly associated with the Cerrado biome [14, 15] and is widely distributed in southeastern and central Brazil [1]. It can also be found in degraded areas of the Atlantic forest [2] and, as a species that benefits from anthropogenic factors, it may frequently be found close to human settlements and riparian forests [16], as well as in overgrown bushes and shrubs in mixed horse pastures [17].

Oliveira et al. [18] compared present-day data on *A. sculptum* and its potential future distribution and concluded that the areas suitable for this tick population may decrease under the current climate change trends. However, these authors warned that making predictions on the expansion of vector populations is problematic given that their response and adaptability to novel climates and warming conditions remain unknown.

### Hosts

**Fig. 2:**
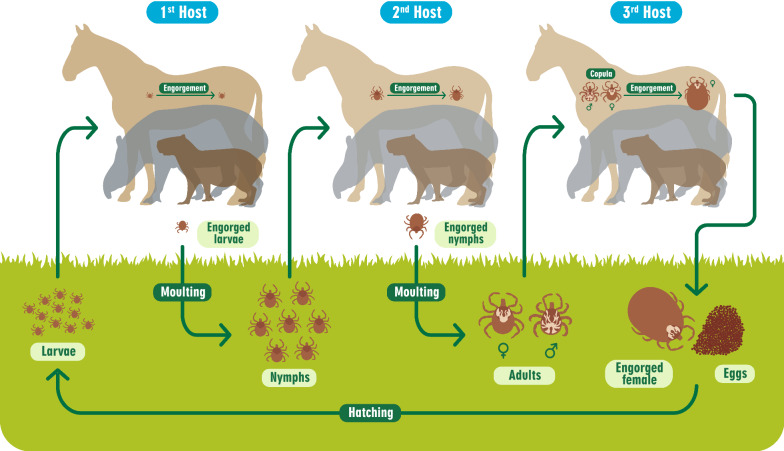
Life-cycle of *Amblyomma sculptum*, a three-host ixodid tick

*Amblyomma sculptum* displays low host specificity, and larvae and nymphs in particular are often found on several animal species, including humans [2, 4]. Although horses (*Equus caballus*), capybaras (*Hydrochoerus hydrochaeris*) and tapirs (*Tapirus terrestris*) are considered to be the most common primary hosts of all parasitic stages of *A. sculptum* in Brazil [2, 4] (Fig. 2), other animals, including peccaries ( *Pecari tajacu*,* Tayassu pecari*), anteaters (*Tamandua tetradactyla*, *Myrmecophaga tridactyla*), armadillos (*Cabassous unicinctus*, *Dasypus novemcinticus*, *Euphractus sexcinctus*), feral and domestic pigs (*Sus scrofa)*, carnivores (*Cerdocyon thous*, *Eira barbara*, *Leopardus pardalis*, *Panthera onca*, *Procyon cancrivorus*), monkeys (*Alouatta guariba*), omnivores (*Didelphis albiventris*, *Nasua nasua*), rodents (*Cuniculus paca*, *Dasyprocta azarae*), deer (*Blastocerus dichotomus*,* Mazama americana*, *Mazama gouazoubira*), reptiles (*Pseudoboa nigra*, *Salvator merianae*), birds (*Amazona aestiva*, *Ara ararauna*, *Aramus guarauna*, *Asio clamator*, *Buteo brachyurus*, *Caprimulgus parvulus*, *Carcara plancus*, *Cariama cristatai*, *Coragyps atratus*, *Tyto furcata*) and cattle (*Bos taurus*, *Bos indicus*) have been reported to be infested by adults and nymphs [19–26]. Humans can also be attacked by all three active developmental stages [4], but more commonly by subadults (larvae and nymphs) than by adults [27]. In three seasonal dynamic studies [14, 28, 29], horses were reported to be frequently parasitized, and tick counts were performed on these animals. The presence of cattle, dogs, marsh deer, capybaras, small rodents, birds, opossums, tapirs, monkeys, coatis, agoutis and several other wild animals were reported in the areas where some of the other studies were conducted (Table 1). These other animals may have been hosts and, therefore, may have contributed to maintenance of this tick species in the area. Nonetheless, 15 (88.2%) of the 17 seasonal dynamic studies included in this systematic review reported the presence of either horses or capybaras, or both, as preferential hosts that were probably responsible for the high quantities of *A. sculptum* in the environment. Interestingly, although cattle have been considered to be major hosts for members of the *A. cajennense* species complex (e.g. *A. mixtum*,* A. patinoi*,* A. tonelliae*) in other South American countries [1], cattle as a host species might not be able to sustain *A. sculptum* populations [23, 30]

**Table 1:** Characteristics of the locations described in the seasonal dynamic studies (between 1990 and 2022) on *Amblyomma sculptum* included in this systematic review

State/provence and country	Area characteristics	Animal presence		Occurrence of spotted fever *Rickettsia* or cases of BSF	References
		Domestic	Wild		
Rio de Janeiro, Brazil	Pastures of 2 farms located in distinct municipalities	Cattle and horses	Information not provided	Information not provided	Souza [43]
São Paulo, Brazil	Pastures of 3 farms in the municipality of Pedreira	Cattle, dogs and horses	Capybaras, small rodents and opossums	Endemic area for BSF	Lemos et al. [31]
Minas Gerais, Brazil	Pastures of an extensive production horse farm	Horses	Information not provided	Information not provided	Oliveira et al. [32]
São Paulo, Brazil	Experimental veterinary faculty ranch; pasture with forage grasses, several bushes and shrub species	Cattle and horses	Information not provided	Information not provided	Labruna et al. [28]
Minas Gerais, Brazil	Pastures of an extensive production horse farm	Horses	Information not provided	Information not provided	Oliveira et al. [29]
São Paulo, Brazil	Farm with remnants of riparian forest	Information not provided	Capybaras	Endemic area for BSF	de Souza et al. [16]
São Paulo, Brazil	Flooded or prone to flooding marsh areas and forest patches	Horses, cattle and dogs	Marsh deer, capybaras, small rodents, birds, opossums and tapirs	Information not provided	Szabó et al. [14]
Paraná, Brazil	Municipal park in urban perimeter	Information not provided	Capybaras, monkeys, opossums, coatis, agouti and birds	No confirmed case of BSF, but possible circulation of spotted fever *Rickettsia* in park workers	Toledo et al. [38]
Minas Gerais, Brazil	Experimental farm floodplain pasture	Cattle, horses and dogs	Capybaras	One confirmed case of BSF	Guedes and Cerqueira Leite [34]
Minas Gerais, Brazil	Cerrado reserve neighboring agricultural fields	Occasionally, dogs and cattle	Sighting of > 24 different mammal species, including peccaries and capybaras	Information not provided	Veronez et al. [15]
São Paulo, Brazil	Six different areas with characteristics not provided	Horses in 3 areas	Capybaras in all areas	Confirmed cases of BSF	Brites-Neto et al. [46]
Jujuy, Argentina	Private reserve in rural perimeter	Information not provided	Information not provided	Information not provided	Tarragona et al. [7]
Minas Gerais, Brazil	National park and in natural areas from neighboring properties	Cattle, dogs and horses	Capybaras	Information not provided	Szabó et al. [39]
Minas Gerais, Brazil	Natural national park	Cattle, horses and dogs	Rich in large-, medium- and small-sized mammals	Rickettsial infection in ticks and positive rickettsial serological analyses in small mammals	Barbieri et al. [35]
Pernambuco, Brazil	Rural areas with remnant forest patches and grazing pastures	Horses and dogs	Capybaras in low abundance, small-sized rodents and marsupials	Information not provided	Dantas-Torres et al. [36]
Goiás, Brazil	University farm set in urban area with riparian forest, flooded areas and forest patches. Grazing pastures and crop fields	Cattle and dogs	Capybaras and coatis	Notified BSF cases	de Paula et al. [37]
Mato Grosso do Sul, Brazil	Two permanent preservation areas with the presence of Cerrado vegetation: one located on a farm owned by Embrapa Beef Cattle, and the second belonging to the State University of Mato Grosso do Sul	Cattle	Not specified but the presence of wild animal trails is mentioned	Information not provided	Garcia et al. [40]

*BSF* Brazilian spotted fever

### Locations of studies

Several studies on the seasonal and population dynamics of *A. sculptum* have been conducted (Fig. 1b) in southeastern Brazil [14, 16, 28, 29, 31–35]. In comparison, fewer studies, as shown in Table 2, refer to northeastern Brazil [36], central-western Brazil [37], southern Brazil [38] and northwestern Argentina [7].

**Table 2:** Seasonal dynamic studies on *Amblyomma sculptum* (between 1990 and 2022) and their methodologies

Biome	State/provence and country	Duration	Collection frequency	Methodology	References
Tropical and Subtropical Moist Broadleaf Forest	Rio de Janeiro, Brazil	2 years (November 1986–October 1988)	Every 14 days (specified only for tick count on horses)	Cloth dragging; cloth trap; tick count on horses	Souza [43]
Tropical and Subtropical Moist Broadleaf Forest	São Paulo, Brazil	1 year (October 1993–October 1994)	Monthly	Removal of feeding ticks from their hosts (human, domestic and wild animals); flagging	Lemos et al. [31]
Tropical and Subtropical Moist Broadleaf Forest	Minas Gerais, Brazil	2 years (July 1995–July 1997)	Every 14 days (between 10 and 12 a.m.)	Cloth dragging; CO_2_ traps (dry ice)	Oliveira et al. [32]
Tropical and Subtropical Grassland Savanna	São Paulo, Brazil	2 years (October 15, 1997 to September 29, 1999)	Every 14 days	Tick count on 10 horses	Labruna et al. [28]
Tropical and Subtropical Grassland Savanna	Minas Gerais, Brazil	2 years (July 1995–July 1997)	Every 14 days	Tick count on 20 horses	Oliveira et al. [29]
Tropical and Subtropical Moist Broadleaf Forest	São Paulo, Brazil	2 years (November 2000–October 2002)	Every 15 days	CO_2_ traps (dry ice) in patches of riparian forest for 1 h/day	de Souza et al. [16]
Tropical and Subtropical Moist Broadleaf Forest	São Paulo, Brazil	22 months (January 2002–November 2003	Approximately every 3 months (8 collections per season)	CO_2_ traps (dry ice); cloth dragging; visual search; inspection of 16 horses	Szabó et al. [14]
Tropical and Subtropical Moist Broadleaf Forest	Paraná, Brazil	1 year (August 2006–July 2007)	Monthly	Cloth dragging; CO_2_ traps (dry ice)	Toledo et al. [38]
Tropical and Subtropical Moist Broadleaf Forest	Minas Gerais, Brazil	2 years (May 2006–April 2008)	Every 14 days	Cloth dragging; CO_2_ traps (one with dry ice and two with chemicals)	Guedes and Cerqueira Leite [34]
Tropical and Subtropical Grassland Savanna	Minas Gerais, Brazil	2 years (June 2006–December 2008)	Every 3 months (13 collections per season)	Cloth dragging (diurnal and nocturnal); CO_2_ traps (dry ice); visual search; domestic animal sampling; tree and nest sampling	Veronez et al. [15]
Tropical and Subtropical Grassland Savanna and Moist Broadleaf Forest	São Paulo, Brazil	1 year (July 2009–June 2010)	Monthly	CO_2_ traps (dry ice)	Brites-Neto et al. [46]
Tropical and Subtropical Grassland Savanna	Jujuy, Argentina	2 years (October 2013–October 2015)	Monthly	Cloth dragging;	Tarragona et al. [7]
Tropical and Subtropical Grassland Savanna	Minas Gerais, Brazil	2 years (April 2007–November 2009)	Every 3 months (8 collections per season)	Cloth dragging; CO_2_ traps; visual search; domestic animal sampling	Szabó et al. [39]
Tropical and Subtropical Grassland Savanna	Minas Gerais, Brazil	21 months (May 2012–February 2014)	Approximately every 3 months (1 expedition per season = 8 expeditions in total)	CO_2_ traps (dry ice); scarce cloth dragging	Barbieri et al. [35]
Tropical and Subtropical Moist Broadleaf Forest	Pernambuco, Brazil	2 years (January 2015–December	Monthly	CO_2_ traps (dry ice)	Dantas-Torres et al. [36]
Tropical and Subtropical Grassland Savanna	Goiás, Brazil	2 years (January 2018–December 2019)	Every 14 days	Cloth dragging; visual search	de Paula et al. [37]
Tropical and Subtropical Grassland Savanna	Mato Grosso do Sul, Brazil	2 years (February 2014–December 2015)	Once every season, specifically February, May, August and December	Cloth dragging; CO_2_ traps (dry ice); visual search	Garcia et al. [40]

It is important to highlight that six (35.3%) of the seasonal dynamic studies were near to or in areas with notified or confirmed cases of human rickettsiosis, or with confirmed circulation of *Rickettsia* spp. (Table 1). Moreover, among the 16 studies conducted in Brazil, 12 (75%; published between 1990 and 2022) were concentrated in its southeastern region. This concentration may also be correlated with the higher number of BSF cases that were notified or confirmed each year in this region. The southeastern region encompasses the states of Minas Gerais, Rio de Janeiro, São Paulo and Espírito Santo, where most of the cases of BSF have been reported, with fatality rates of approximately 50% [9]. On the other hand, four (23.5%) of the most recent studies were published between 2018 and 2022 in areas that had not previously been studied, such as the northeastern and central-western regions of Brazil and the only study in Argentina. These studies thus emphasize the distribution and importance of *A. sculptum* in other regions and countries and, consequently, its possible role as a vector of tick-borne diseases in other localities. It is also worth noting that while Szabó et al. [39] and Garcia et al. [40] published their findings in 2018 and 2022, respectively, they conducted their respective studies between 2007 and 2009 [39] and between 2014 and 2015 [40].

Of the 17 studies, eight (47.0%) were conducted in areas of tropical and subtropical moist broadleaf forest biome and eight (47.0%) were conducted in areas of tropical and subtropical savanna grassland biome; one (5.9%) was conducted in areas of both biomes (Fig. 1b; Table 2). These choices of study sites emphasize the wide distribution of *A. sculptum* in two biomes that are very widely scattered throughout South America and encompass a large portion of Brazil [41]. *Amblyomma sculptum* has even been found in degraded areas of the Atlantic Forest biome with abiotic conditions closer to those of the Cerrado (savanna) biome [2], suggesting that the distribution of this tick has expanded along with the degradation of the Atlantic Forest.

### Methodologies used for *A. sculptum* collection

In the 17 studies, various methodologies, including carbon dioxide (CO_2_) traps, flagging, dragging, tick counting on animals and visual searches, were used separately or simultaneously to determine the seasonal dynamics of *A. sculptum*.

It is important to maintain consistency in implementing these methods in all expeditions, not only regarding the techniques but also in relation to maintaining controlled intervals between expeditions and always executing them over similar time periods with the same numbers of team members, traps and cloth. This minimizes the interference of the method on the results [14].

In addition, it is important to continue to collect samples for a minimum of 24 months (2 years), as was done in most of the published studies included in this review (12/17; 70.6%; Table 2). The time interval contributes to a higher reliability for the results, given that repetition of life-cycle characteristics over consecutive years ensures that seasonal aspects are observed. However, the frequency of expeditions may vary: seven (41.2%) of the studies chose an interval of 14–15 days, while five (29.4%) collected samples monthly, and five (29.4%) collected samples every 3 months (Table 2).

#### Dragging and flagging

Free-living active stages of *A. sculptum* (host-questing larvae, nymphs and adults) can be captured by techniques known as drag or flag sampling using a white cloth [42]. Several studies on the seasonal or population dynamics of *A. sculptum* [7, 14, 15, 31, 32, 34, 35, 37–40, 43] (12/17; 70.6%) adopted modified versions of this technique (Table 2). During the dragging technique, the cloth, which is attached to one or two metal or wooden rods/poles, is kept close to the vegetation and dragged a short distance over the ground, over plant litter and vegetation, before pausing for inspection, thus minimizing loss of the captured ticks. Similarly, during flagging, the cloth is kept close to slightly higher vegetation while attached to one metal or wooden pole/rod and is also used over short distances before close inspection. The size of the cloth can vary, although it should remain between 1.0–1.5 and 0.7–0.9 m (Additional file 1: Table S1). It is important to note that the success of this method may be diminished when performed during the rainy season, in areas with dense vegetation or in areas prone to flooding [14].

A comparison between flagging and dragging was conducted by Dantas-Torres et al. [44] in southern Italy involving other tick species. These authors observed that more adult ticks were collected during flagging on animal and man-made trails, while dragging was more efficient when used in grassland. However, the efficiency of each method varied according to the specific species collected. As a result of these findings, they suggested that field studies should always take into consideration factors linked to tick behavior, habitat characteristics and climate before choosing and using the techniques. These factors are directly associated with variations in collection and if the collecting methods are not carefully chosen and implemented, they may result in misleading conclusions.

#### CO_*2*_ traps

Traps using CO_2_ as a chemical attractant (CO_2_-baited traps) [45] are commonly used to capture nymphs and adults since CO_2_ is considered to be a universal attractant for hematophagous insects and is efficient for attracting *A. sculptum* ticks [15, 35]. White cloths (average size: 1 × 1 m) with double-sided adhesive tape placed along all four edges are placed in different parts of a pasture near riparian forest or in patches of the pasture that remain shaded from direct sunlight during the mornings. Dry ice cubes, in amounts ranging from 500 g to 1 kg, are placed inside thermal boxes containing holes, which are then placed in the middle of the cloths; alternatively, the dry ice cubes are placed directly in the middle of the cloths without containers. The length of exposure may vary but is generally between 30 min and 2 h (Additional file 1: Table S1). Although the basic principle of the technique is as described here, many variations exist and each of the studies assessed in this review implemented its own adaptation. After the sample collection period, the cloths are removed and kept individually in plastic bags until the ticks that became stuck to the adhesive are collected, identified and counted [16, 32, 34]. This technique was used by Oliveira et al. [32], de Souza et al. [16], Szabó et al. [14], Toledo et al. [38], Guedes and Cerqueira Leite [34], Veronez et al. [15], Brites-Neto et al. [46], Szabó et al. [39], Barbieri et al. [35] and Dantas-Torres et al. [36] (Table 2).

In comparing visual searches, CO_2_ traps, dragging and plant litter examination, Ramos et al. [47] described CO_2_ traps attracting ticks from around the trap without pinpointing where they were originating from. This method was applied in 64.7% (11/17) of the studies, the same proportion as cloth dragging. Although highly efficient, CO_2_ traps have proved to be expensive and troublesome, especially because of lack of availability in smaller cities and rural areas and because of problems regarding their transportation, storage and durability.

#### Visual search

*Amblyomma sculptum* is a three-host tick (Fig. 2) whose nymphs and adults are known to exhibit more than one strategy for seeking hosts [10], although this behavior has never specifically been studied. They are considered to be extremely fast-moving ticks [14, 15, 47, 48] and have demonstrated a hunter-tick nature, characteristically displaying ambush behavior, such as by awaiting potential hosts while perched on the tips of vegetation [49]. As a host-seeking tick species, therefore, it can be found on the vegetation in forestall phytophysiognomies by riversides that are not flooded and along well-defined animal trails in forest patches frequently used by hosts [47].

Searching for *A. sculptum* in the environment when the tick is displaying host-questing behavior on the tip of vegetation leaves is referred to as the visual search or inspection method. This approach considers the behavioral characteristics of *A. sculptum*, such as its questing nature and the type, location and height of the vegetation on which it is more likely to achieve its aim of finding and attaching to a potential host [14, 50]. Szabó et al. [14], Veronez et al. [15], Szabó et al. [39] and de Paula et al. [37] included this technique in their methodology (Table 2; Additional file 1: Table S1).

Labruna et al. [51] observed *A. sculptum* larvae exhibiting questing behavior on the top of vegetation, at heights ranging from 15 to 50 cm, although an ‘atypical’ height of 185 cm was recorded in the same study. Ramos et al. [49] observed that host-questing *A. sculptum* could be found on vegetation varying in height from a median of 25 cm for nymphs and 30–32 cm for adults. These ticks were mostly observed at plant heights of > 10 or > 15 cm above the ground. Taken together, these results indicate that *A. sculptum* seeks medium- to large-sized hosts, which is corroborated by the rarity of finding this tick parasitizing small rodents in areas where *A. sculptum* is the most abundant tick species in the environment [35, 52].

Atmospheric vapor saturation deficit is a key abiotic factor that also influences vertical movement of ambush ticks on vegetation. Temperature and humidity conditions also play a role in host-seeking activity on the vegetation, such that these ticks may descend to the ground if these two factors are unsuitable [53]. *Amblyomma sculptum* may stop seeking hosts through ambush behavior in shaded areas after the end of the morning hours [49], which emphasizes other observations indicating preference for shaded spaces [3, 15, 17]. Therefore, it is preferable to use visual searches in areas that are shaded and more humid, during the earlier hours of the day, with the aim of collecting the largest quantities of ticks possible. Furthermore, despite not being properly demonstrated through comparative studies, sampling length in time and space may influence the results on tick collected, especially depending on specific tick behavior. For example, if a collector stands for a longer period in the same space, then more CO_2_ will be exhaled, thus serving as more stimuli for ticks exhibiting host-seeking behavior. However, if the collector treks for a longer distance by staying shorter periods of time in each place, some of the ticks attracted to the spot might be missed. For this reason, some researchers opt for one collector walking a few minutes ahead while the other follows shortly after to observe the ticks that might have been stimulated by the first collector.

Although Terassini et al. [50] did not collect *A. sculptum*, they compared the visual search method with dragging and found that visual searches were more suitable for collecting adult ticks, while dragging was better for maximizing collection of immature stages. According to their findings, these two techniques can be performed by the same investigator simultaneously, while walking along an animal trail.

After comparing different methods, Ramos et al. [47] reported that visual searches led to the collection of larger quantities of adult ticks while also providing information on tick-questing height. Their data also indicated that using a single technique did not provide a representative sample of the tick fauna and, therefore, that multiple methods should be used.

While visual searches are by far the simplest and most inexpensive method for collecting *A. sculptum*, they were only used in five of the 17 studies included in this review (29.4%). Visual searches are more effective for detecting adult stages, which are easier to notice than the smaller stages. However, the efficacy of visual searches is extremely dependent on the observer’s attention and knowledge of where on the vegetation, and how high, they should inspect.

#### Remarks on collecting free-living ticks

Oliveira [54] compared the efficiency of dragging and CO_2_ traps (dry ice) for collecting larvae, nymphs and adults of *A. sculptum,* and concluded that dragging was more efficient for capturing larvae, whereas CO_2_ traps were more efficient for capturing nymphs and adults. This author argued that these results were related to the limited horizontal active displacement of larvae (i.e. difficult to reach a CO_2_ trap) while the large size of post-larval stages (especially adults) accounted for the lower efficiency of dragging. Specifically, many captured specimens were dislodged from the white cloth in contact with vegetation during flagging before the white cloth was checked by the researcher*.*

Visual search for ticks on vegetation, CO_2_ traps, dragging and plant litter examination were compared in another study in an area with *A. sculptum* predominance [47], in which all four techniques were indicated for hunting and ambushing ticks. These techniques can usually be used in combination, as one technique complements the other. However, dragging was determined to be the most efficient method, especially for nymphs. In addition, dragging is a lower-cost method, and its materials are easy to obtain and transport. These reasons may have contributed to dragging being one of the most commonly used methods, along with CO_2_ traps, since 64.7% (11/17) of the studies used cloth dragging.

The efficiency of any method for collecting free-living stages of *A. sculptum* must always consider the environmental preferences of this tick species. Labruna [55] and Luz et al. [56] reinforced earlier circumstantial evidence obtained through field and laboratory observations, inferring that high humid, dense forests (e.g. Amazon primary forest) or flooded soils might not be suitable environments for *A. sculptum* [55, 56]. This may explain why this species is known to be more abundant in open areas with shrubs or forest fragments with areas prone to drought, than in areas with seasonal flooding [14, 35, 57]. Likewise, these results corroborate observations made by Szabó et al. [14] of significantly higher numbers of *A. sculptum* found in forest patches than in nearby marshy areas. Barbieri et al. [35] observed an abundance of this tick species in Cerradão (woodland with crown cover of 50–90%, made up of trees, often 8-12 m high) and gallery forest areas, which present denser canopies and thicker leaf litter—attributes that are important in providing optimal microclimate conditions. Estrada-Peña et al. [58] reported that *A. sculptum* showed a preference for environments with monthly average temperatures mostly raging between 21 ºC and 27 ºC. Ten (58.8%) of the seasonal dynamic studies were conducted in locations in farm pastures, six (35.3%) in nature reserves and/or nature parks; the authors of one study (5.9%) did not specify the location (Table 1). Areas containing shrubs, bushes and/or fragments of natural forests, as well as both wild and domestic animals, thus form ideal sites for studies involving *A. sculptum.*

Although the studies mentioned above have provided important data for improving our understanding of the preferences of *A. sculptum*, further research is needed to better comprehend the field conditions that enable its survival and reproduction. Such studies will provide concrete maximum and minimum relative humidity and temperature values, as well as other factors influencing its distribution and prevalence*.*

#### Tick counting on animals

Evaluation of the numbers of ticks parasitizing specific hosts is another technique that is used to obtain data on *A. sculptum* populations. This methodology was described and tested by Oliveira [54]. It can be used for collecting all three parasitic stages of *A. sculptum*. Studies conducted by Labruna et al. [28] and Oliveira et al. [29] (Table 2) used modifications of this technique. In their modified method, ticks are counted every 14 days, preferably in the morning, on the entire left side of horses, the preferred host for this tick species, kept on naturally infested pastures. These animals are selected in descending order based on the number of infested adult stages, with the aim of reducing the possible effects of natural individual resistance to *A. sculptum*. The total number of each tick stage counted is then doubled to estimate the overall number of ticks on each animal. Adult male and females are counted individually without being removed; larvae and nymphs are counted after being removed from the host by using a soft and flexible rubber scraper, which detaches only engorged immature specimens. This technique is facilitated by the fact that the neck, shoulder and thoracic region are predilection sites for larvae and nymphs of *A. sculptum* on horses [54]. In an attempt to prevent injurious levels of *A. sculptum* infestations, Oliveira et al. [29] divided the sampled horses into four groups and sprayed only one group with a low residual-commercial acaricide after each counting session, alternating the group sprayed in consecutive sessions.

Nonetheless, tick counts on the animals in the above studies differ from those obtained in the methods used by Szabó et al. [14, 39] and Veronez et al. [15, which are known as tick sampling on animals. This method consists of collecting a sample of ticks from domestic animals, such as dogs, horses or cattle, from neighboring areas. This type of methodology does not require that the same animals be kept in the study: some may leave the farm and new animals may arrive. Also, there is no counting of ticks on an entire side of an animal; instead, as many ticks as possible are collected, which may or may not be done within a predetermined time interval.

Although horses are primary hosts, tick counts on these animals were used in only 17.6% (3/17) of the seasonal dynamic studies. This was probably due to insufficient numbers of horses in other study locations, even though horses were reported in 11 of the 17 (64.7%) studies. Moreover, although tick counting on other hosts is possible, it becomes unfeasible due to the difficulty and costs of capturing, containing and handling wild animals, especially other primary hosts species, such as capybaras and tapirs.

## Seasonal dynamics of *A. sculptum*

Overall, the studies carried out over the last two decades have all reported the conclusion that *A. sculptum* completes one generation per year. Although all three parasitic stages are generally found throughout the year, seasonal preferences are demonstrated, depending on the climatic characteristics of each region. Also, despite the similar results that have been found between the southern, southeastern and central-western regions of Brazil and the distinct results from the northeastern region, as discussed below, the seasonal dynamics of this tick species should be related to distinct latitudes (i.e. distinct photoperiod range throughout the year) and differences in seasonal weather patterns. For example, all studies in the southern, southeastern and central-western regions were from areas characterized by a cool and dry winter, and a hot and rainy summer, in contrast to the single study in the northeastern region [36] where the weather is warm throughout the year and the dry season is in the summer. However, the authors of the latter study caution the reader and highlight possible biased results due to the relatively low number of ticks collected.

It is important to note that up until 2014, published studies on *A. sculptum* referred to *A. cajennense.* In addition, since there is no taxonomic key for Brazilian *Amblyomma* larvae, larvae were identified only to the genus level [59]. Nymphs were identified only to genus level after 2010 [60, 61] and adults were identified as *A. sculptum* only after 2014 [1, 60–62]. Therefore, most of the information published on this topic involves larvae and nymphs of *Amblyomma* spp. without identification to the species level.

**Fig. 3:**
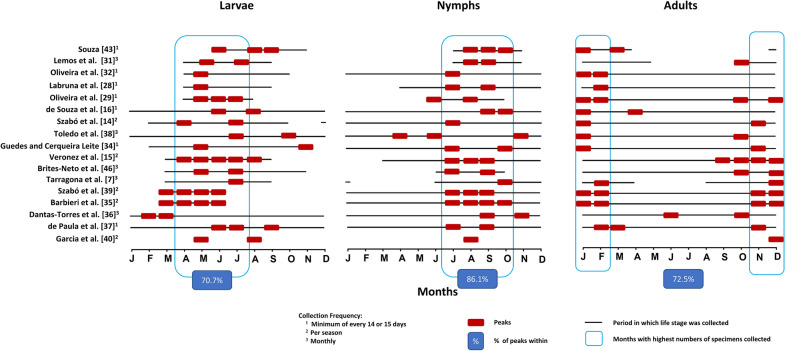
Graph demonstrating the months in which the peak numbers of each free-living stage of *Amblyomma sculptum* occurred, as described in the respective published study

While different methodologies were applied in these seasonal studies on *A. sculptum*, among which a few consisted of tick counting on horses [14, 28, 29, 43], similar results were observed (Fig. 3). Peaks in the number of larvae were observed during mid-autumn and early winter, and peaks in the number of nymphs were observed during winter and early spring; the number of adults increased during the spring and peaked during the summer in most locations.

It can also be noted that the use of a combination of different sampling methods can potentially increase the number of ticks counted during a study period, as seen in de Paula et al. [37], who reported a total of 117,685 ticks using cloth dragging and visual searches. The use of only one method, if the choice is appropriate for the species targeted, has proved to be equally if not more efficient. Such was the case of tick counting on horses, with 340,200 counted by Labruna et al. [28] and 50,117 counted by Oliveira et al. [29]. These results contribute towards emphasizing the importance of knowing the behavioral characteristics and preferences of the species under study. This knowledge is powerful for guiding researchers towards the correct path regarding which methodology to use and how, where and for how long to apply it.

### Larvae

Larvae are most commonly found in slightly higher quantities between April and November, as shown in Additional file 2: Table S2 [14, 16, 28, 29, 31–36, 38]. However, 70.7% of the larval peaks were found to be concentrated between April and July, i.e. during mid-autumn and early winter, as shown in Fig. 3. These may have occurred because of larval diapause or tick dormancy behavior, which precedes the onset of unfavorable environmental conditions and is primarily regulated by photoperiod [63].

Two types of diapause have been described in ticks: behavioral and morphogenic. The former consists of suppression of host-seeking activity in unfed stages or host feeding on hosts; the latter consists of delayed embryogenesis, metamorphosis of larvae and nymphs, or oogenesis of engorged females [63].

**Fig. 4:**
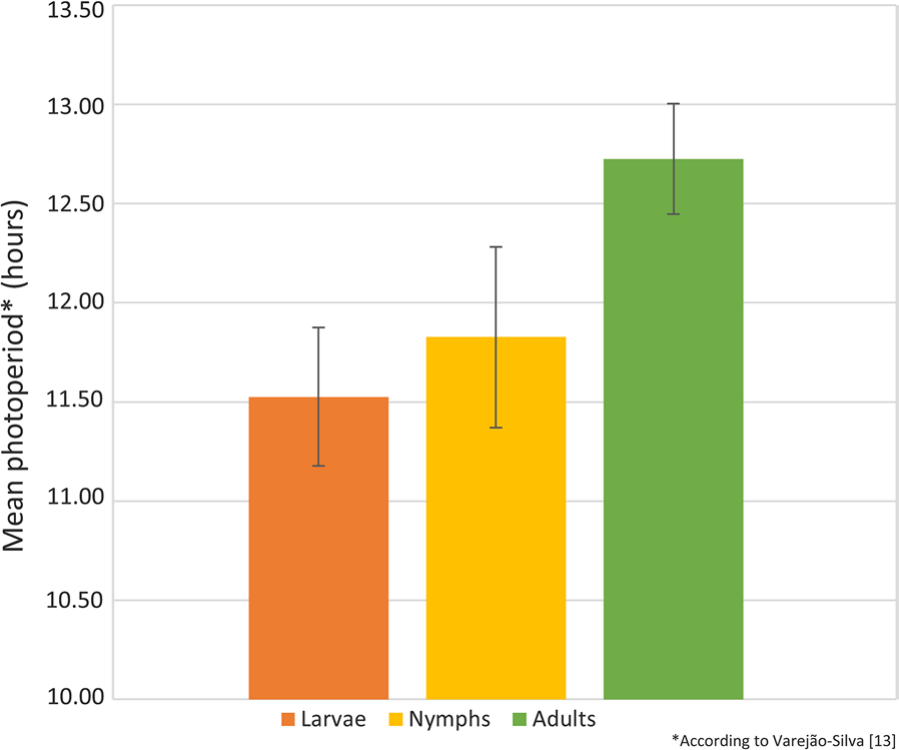
Mean photoperiod hours, compiled from all published data reported in this review, of when the highest numbers of each stage (larvae, nymphs and adults) of *Amblyomma sculptum* were collected and counted

Behavioral diapause has been described for *A. sculptum* larvae (published as *A. cajennense*) in Brazil [64, 65]. In this case, the diapause suppresses larval host-seeking activity during the summer in order to synchronize the life-cycle for the future adult ticks to quest chiefly during the upcoming rainy season (spring–summer months). This allows females to lay eggs during the periods with the most humid climate and highest vegetation, thus favoring egg incubation, which is the stage that is most sensitive to abiotic factors [65]. This larval diapause occurs when days are longer, with > 12 h of daylight, as shown in Fig. 4 [13]. In all seasonal dynamic studies, the mean photoperiod value was around 11.5 h for the months during which the highest numbers of larvae were counted, coinciding with a mild and dry weather. It would therefore appear that larval behavioral diapause regulates the one-generation pattern of *A. sculptum* in southeastern Brazil, as demonstrated by Labruna et al. [65] in a field study in Pirassununga County, São Paulo state, southeastern Brazil.

The results of one study differed from those of the other studies. Dantas-Torres et al. [36] showed larval peaks in the early months of the year, concentrated between February and March. This divergence may have been the result of distinct seasonal weather patterns observed in the region of that study, compared with the other studies. For example, in the northeastern region, the dry period is concentrated in the summer, while it occurs mostly during the winter in the central-western and southeastern regions [36]. In addition, according to these authors, this distinct result may have been biased or masked by the low numbers of ticks collected during their experiment.

### Nymphs

Higher numbers of nymphal activity were mostly reported to occur from May to November, only a few weeks after the increase in the quantities of larvae (Additional file 2: Table S2). However, 86.1% of the peaks in and predominance of nymphs were concentrated between July and October, during the winter and early spring [7, 14–16, 28, 29, 31, 32, 34–40, 43, 46], as shown in Fig. 3. The largest numbers were seen during days with a shorter daylight period, i.e. with mean photoperiod < 12 h (overall mean: 11.8 h) [13], as seen in Fig. 4. Similar to the results for larvae, a single experiment reported different patterns for nymphs [36], with the probable causes of those variations reported to be distinct weather and photoperiod patterns and the low numbers of collected ticks, as for larvae [36].

It has been observed for decades that most of the human cases of tick-borne rickettsiosis (chiefly caused by *R. rickettsii*) occurs during the second semester of year, especially during midwinter to spring [66]. This observation has led authors to infer that the nymphal stage of *A. sculptum* is the most important stage for transmitting *R. rickettsii* to humans, emphasizing the need to expand surveillance measures during the months of nymph abundance. It is noteworthy that in a 2-year study on human parasitism during a survey on ticks in a reserve of the Brazilian savanna [67], among all of the 439 ticks collected from human hosts, *A. sculptum* nymphs were the most abundant and the ticks most frequently biting humans.

### Adults

Larger numbers of adults were collected between September and April (Additional file 2: Table S2), with 72.5% of the peak numbers occurring between November and February, i.e. during the spring and summer, as shown in Fig. 3 [7, 14–16, 28, 29, 31, 32, 34, 35, 37–40, 43, 46]. Moreover, this stage is more active in periods with longer days, i.e. with > 12 h of daylight (Fig. 4) [13], and as already mentioned, temperature and humidity influence host-seeking activity, as well as the descent to the ground and oviposition [49, 53].

A similar seasonal pattern has been described for other *Amblyomma* species of the *Amblyomma cajennense* complex, such as *A. tonelliae* [7] and *A. mixtum* (published as *A. cajennense*) [68]. These latter two species also have a life-cycle consisting of one generation per year, with larvae and nymphs predominating during the dry season and adults predominating during the rainy season. However, *A. mixtum* showed a longer period of larval and nymphal activity during the year [68].

## Conclusion

It is evident that several major studies have already been conducted on the populational and seasonal dynamics of *A. sculptum*, encompassing various environments where this tick is found, both in preserved areas as well as those anthropized. Thus, much important information on this subject has been surveyed. Despite the differences in rainfall volumes, temperature, seasonal characteristics, distribution and availability of hosts and phytophysiognomy in these studies, *A. sculptum* always presented a one-generation-per-year cycle. Given this tick’s wide distribution, further studies carried out in unexplored areas is necessary to fully describe its seasonal and ecological profiles, especially in northeastern Brazil, where photoperiod variations are less evident throughout the year.

## Supplementary Information

**Additional file 1: Table S1.** Additional data on collection methodology applied by *Amblyomma sculptum* seasonal dynamic studies between 1990 and 2022.

**Additional file 2: Table S2.** Results from *Amblyomma sculptum* seasonal dynamic studies between 1990 and 2022 and mean photoperiod (PP: daylight length in hours) at the time of highest availability of each active stage.

## Data Availability

All data generated or analyzed during this study are included in this published article and its additional files.

## References

[1] Nava S, Beari L, Labruna MB, Cáceres AG, Mangold AJ, Guglielmone AA (2014). Reassessment of the taxonomic status of *Amblyomma cajennense* (Fabricius, 1787) with the description of three new species, *Amblyomma tonelliae* n.sp., *Amblyomma interandinum* n.sp. and *Amblyomma patinoi* n.sp., and reinstatement of *Amblyomma mixtum* Koch, 1844, and *Amblyomma sculptum* Berlese, 1888 (Ixodida: Ixodidae). Ticks Tick Borne Dis..

[2] Martins TF, Barbieri ARM, Costa FB, Terassini FA, Camargo LMA, Peterka CRL (2016). Geographical distribution of Amblyomma cajennense (sensu lato) ticks (Parasitiformes: Ixodidae) in Brazil, with description of the nymph of *A. cajennense* (sensu stricto). Parasit Vectors.

[3] Ramos VN, Osava CF, Piovezan U, Szabó MPJ (2014). Ticks on humans in the Pantanal wetlands. Brazil Ticks Tick Borne Dis.

[4] Guglielmone AA, Beati L, Barros-Battesti DM, Labruna MB, Nava S, Venzal JM (2006). Ticks (Ixodidae) on humans in South America. Exp Appl Acarol.

[5] Labruna MB, Krawczak FS, Gerardi M, Binder LC, Barbieri ARM, Paz GF (2017). Isolation of *Rickettsia rickettsii* from the tick *Amblyomma sculptum* from a Brazilian spotted fever-endemic area in the Pampulha Lake region, southeastern Brazil. Vet Parasitol Reg Stud Rep.

[6] Krawczak FS, Nieri-Bastos FA, Nunes FP, Soares JF, Moraes-Filho J, Labruna MB (2014). Rickettsial infection in *Amblyomma sculptum* ticks and capybaras (*Hydrochoerus hydrochaeris*) in a Brazilian spotted fever-endemic area. Parasit Vectors.

[7] Tarragona EL, Sebastian PS, Saracho Bottero MN, Martinez EI, Debárbora VN, Mangold AJ (2018). Seasonal dynamics, geographical range size, hosts, genetic diversity and phylogeography of *Amblyomma sculptum* in Argentina. Ticks Tick Borne Dis.

[8] Peckle M, Pires MS, Dos Santos TM, Roier EC, da Silva CB, Vilela JA (2013). Molecular epidemiology of *Theileria equi* in horses and their association with possible tick vectors in the state of Rio de Janeiro, Brazil. Parasitol Res.

[9] Szabó MPJ, Pinter A, Labruna MB (2013). Ecology, biology and distribution of spotted-fever tick vectors in Brazil. Front Cell Infect Microbiol.

[10] PajuabaNeto AA, Ramos VDN, Martins MM, Osava CF, Pascoal JO, Suzin A (2018). Influence of microhabitat use and behavior of *Amblyomma sculptum* and *Amblyomma dubitatum* nymphs (Acari: Ixodidae) on human risk for tick exposure, with notes on *Rickettsia* infection. Ticks Tick Borne Dis..

[11] QGIS.org. QGIS Geographic Information System. QGIS Association. 2021. http://www.qgis.org. Acessed 21 May 2021.

[12] Adobe Inc. Adobe Illustrator. San Jose: Adobe Inc. 2019. http://www.adobe.com.

[13] Varejão-Silva MA. Meteorologia e climatologia. Versão Digital 2. Recife, Pernambuco. 2006. https://icat.ufal.br/laboratorio/clima/data/uploads/pdf/METEOROLOGIA_E_CLIMATOLOGIA_VD2_Mar_2.

[14] Szabó MPJ, Castro MB, Ramos HGC, Garcia MV, Castagnolli KC, Pinter A (2007). Species diversity and seasonality of free-living ticks (Acari: Ixodidae) in the natural habitat of wild Marsh deer (*Blastocerus dichotomus*) in Southeastern Brazil. Vet Parasitol.

[15] Veronez VA, Freitas BZ, Olegário MMM, Carvalho WM, Pascoli GVT, Thorga K (2010). Ticks (Acari: Ixodidae) within various phytophysiognomies of a Cerrado reserve in Uberlândia, Minas Gerais, Brazil. Exp Appl Acarol.

[16] de Souza SSAL, Souza CE, Rodrigues Neto EJ, Prado AP (2006). Dinâmica sazonal de carrapatos (Acari: Ixodidae) na mata ciliar de uma área endêmica para febre maculosa na região de Campinas, São Paulo. Bras Ciênc Rural..

[17] Labruna MB, Kerber CE, Ferreira F, Faccini JLH, De Waal DT, Gennari SM (2001). Risk factors to tick infestations and their occurrence on horses in the state of São Paulo, Brazil. J Vet Parasitol.

[18] Oliveira SV, Romero-Alvarez D, Martins TF, Santos JPD, Labruna MB, Gazeta GS (2017). *Amblyomma* ticks and future climate: Range contraction due to climate warming. Acta Trop.

[19] Martins TF, Reis JL, Viana EB, Luz HR, Oda FH, Dantas SP (2020). Ticks (Acari: Ixodidae) on captive and free-ranging wild animals in Tocantins State, a Cerrado-Amazon transition region of northern Brazil. Int J Acarology.

[20] Medri ÍM, Martins JR, Doyle RL, Mourão G, Marinho-Filho J (2010). Ticks (Acari: Ixodidae) from yellow armadillo, *Euphractus sexcinctus* (Cingulata: Dasypodidae), in Brazil's Pantanal wetlands. Neotrop Entomol.

[21] Pereira MC, Szabó MPJ, Bechara GH, Matushima ER, Duarte JMB, Rechav Y (2000). Ticks (Acari: Ixodidae) associated with wild animals in the Pantanal region of Brazil. J Med Entomol.

[22] Ramos VN, Piovezan U, Franco AHA, Osava CF, Herrera HM, Szabó MPJ (2014). Feral pigs as hosts for *Amblyomma sculptum* (Acari: Ixodidae) populations in the Pantanal, Mato Grosso do Sul, Brazil. Exp Appl Acarol..

[23] Ramos VN, Piovezan U, Franco AHA, Rodrigues VS, Nava S, Szabó MPJ (2016). Nellore cattle (*Bos indicus*) and ticks within the Brazilian Pantanal: ecological relationships. Exp Appl Acarol.

[24] Witter R, Martins TF, Campos AK, Melo ALT, Corrêa SHR, Morgado TO (2016). Rickettsial infection in ticks (Acari: Ixodidae) of wild animals in midwestern Brazil. Ticks Tick Borne Dis.

[25] Martins TF, Milanelo L, Krawczak FS, Furuya HR, Fitorra LS, Dores FT (2017). Diversity of ticks in the wildlife screening center of São Paulo city, Brazil. Ciênc Rural..

[26] Mendoza-Roldan J, Ribeiro SR, Castilho-Onofrio V, Grazziotin FG, Rocha B, Ferreto-Fiorillo B (2020). Mites and ticks of reptiles and amphibians in Brazil. Acta Trop.

[27] Labruna MB (2009). Ecology of *Rickettsia* in South America. Ann N Y Acad Sci.

[28] Labruna MB, Kasai N, Ferreira F, Faccini JLH, Gennari SM (2002). Seasonal dynamics of ticks (Acari: Ixodidae) on horses in the state of São Paulo, Brazil. J Vet Parasitol.

[29] Oliveira PR, Borges LMF, Leite RC, Freitas CMV (2003). Seasonal dynamics of the Cayenne tick, *Amblyomma cajennense* on horses in Brazil. Med Vet Entomol.

[30] Siqueira SM, Maia RC, Ramos VN, Rodrigues VS, Szabó MPJ (2021). *Rhipicephalus microplus* and *Amblyomma sculptum* (Ixodidae) infestation of nellore cattle (*Bos taurus indicus)* in a farm of the Brazilian Cerrado: seasonality and infestation patterns. Exp Appl Acarol.

[31] Lemos ERSM, Raimundo D, Coura JS, Guimarães MAA, Serra Freire NM, Amorim MG, Giberto S (1997). Epidemiological aspects of the Brazilian spotted fever: seasonal activity of ticks collected in an endemic area in São Paulo, Brazil. Rev Soc Bras Med Trop..

[32] Oliveira PR, Borges LMF, Lopes CML, Leite RC (2000). Population dynamics of free-living stages of *Amblyomma cajennense* (Fabricius, 1787) (Acari: Ixodidae) on pastures of Pedro Leopoldo, Minas Gerais State, Brazil. J Vet Parasitol.

[33] Labruna MB, Terassini FA, Camargo LMA (2009). Notes on population dynamics of *Amblyomma* Ticks (Acari: Ixodidae) in Brazil. J Parasitol.

[34] Guedes E, Leite RC (2008). Dinâmica sazonal de estádios de vida livre de *Amblyomma cajennense* e *Amblyomma dubitatum* (Acari: Ixodidae) numa área endêmica para febre maculosa, na região de Coronel, Pacheco, Minas Gerais. Rev Bras Parasitol Vet.

[35] Barbieri ARM, Szabó MPJ, Costa FB, Martins TF, Soares HS, Pascoli G (2019). Species richness and seasonal dynamics of ticks with notes on rickettsial infection in a Natural Park of the Cerrado biome in Brazil. Ticks Tick Borne Dis.

[36] Dantas-Torres F, Melo MF, Sales K, da Silva FJ, Figueredo LA, Labruna MB (2019). Phenology of *Amblyomma sculptum* in a degraded area of Atlantic rainforest in north-eastern Brazil. Ticks Tick Borne Dis.

[37] de Paula LGF, Zeringóta V, Sampaio ALN, Bezerra GP, Barreto ALG, dos Santos AA, Miranda VC, Paula WVF, Neves LC, Secchis MV, Alves SGA, Sevá AP, Borges LMF, Monteiro C, Dantas-Torres F, Krawczak FS (2021). Seasonal dynamics of *Amblyomma sculptum* in two areas of the Cerrado biome midwestern Brazil, where human cases of rickettsiosis have been reported. Exp Appl Acarol.

[38] Toledo RS, Haydu VB, Tamekuni K, Vidotto O (2008). Seasonal dynamics of *Amblyomma* ticks (Acari:Ixodidae) in an urban Park of Londrina City, Parana, Brazil. Rev Bras Parasitol Vet.

[39] Szabó MPJ, Martins MM, de Castro MB, Pacheco RC, Tolesano-Pascoli GV, Dos Santos KT (2018). Ticks (Acari: Ixodidae) in the Serra da Canastra National Park in Minas Gerais, Brazil: species, abundance, ecological and seasonal aspects with notes on rickettsial infection. Exp Appl Acarol.

[40] Garcia MV, Zimmermann NP, Rodrigues VS, Aguirre AAR, Higa LOS, Matias J (2022). Tick fauna in non-anthropogenic areas in Mato Grosso do Sul, Brazil, with the presence of the *Rickettsia parkeri* strain Atlantic rainforest in *Amblyomma ovale*. Ticks Tick Borne Dis..

[41] Olson DM, Dinerstein E, Wikramanayake ED, Burgess ND, Powell GVN, Underwood EC (2001). Terrestrial ecoregions of the world: A new map of life on Earth: A new global map of terrestrial ecoregions provides an innovative tool for conserving biodiversity. Bioscience.

[42] Sonenshine DE, Atwood EL, Lamb JTJR (1966). The ecology of ticks transmitting Rocky Mountain Spotted Fever in a study area in Virginia. Ann Entomol Soc Am.

[43] Souza AP. Variação populacional dos principais ixodídeos parasitas de bovinos e equinos em diferentes condições de manejo, nos municípios de Paracambi e Itaguaí no estado do Rio de Janeiro. Doctoral thesis. Rio de Janeiro: Universidade Federal Rural do Rio de Janeiro; 1990 (in Portuguese).

[44] Dantas-Torres F, Lia RP, Capelli G, Otranto D (2013). Efficiency of flagging and dragging for tick collection. Exp Appl Acarol.

[45] Wilson JG, Kinzer DR, Sauer JR, Hair JA (1972). Chemo-attraction in the lone star tick (Acarina: Ixodidae). I. Response of different developmental stages to carbon dioxide administered via traps. J Med Entomol.

[46] Brites-Neto J, Nieri-Bastos FA, Brasil J, Duarte KMR, Martins TF, Veríssimo CJ (2013). Environmental infestation and rickettsial infection in ticks in an area endemic for Brazilian spotted fever. Rev Bras Parasitol Vet.

[47] Ramos VN, Osava CF, Piovezan U, Szabó MPJ (2014). Complementary data on four methods for sampling free-living ticks in the Brazilian Pantanal. Rev Bras Parasitol Vet.

[48] Szabó MPJ, Labruna MB, Garcia MV, Pinter A, Castagnolli KC, Pacheco RC (2009). Ecological aspects of the free-living ticks (Acari: Ixodidae) on animal trails within Atlantic rainforest in south–eastern Brazil. Ann Trop Med Parasitol.

[49] Ramos VN, Osava CF, Piovezan U, Szabó MPJ (2017). Ambush behavior of the tick *Amblyomma sculptum* (*Amblyomma cajennense* complex) (Acari: Ixodidae) in the Brazilian Pantanal. Ticks Tick Borne Dis..

[50] Terassini FA, Barbieri FS, Albuquerque S, Szabó MPJ, Camargo LMA, Labruna MB (2010). Comparison of two methods for collecting free-living ticks in the Amazonian forest. Ticks Tick Borne Dis.

[51] Labruna MB, Pinter A, Castro MB, Castagnolli KC, Szabó MJP (2002). Some records on host questing behavior of *Amblyomma cajennense* (Acari: Ixodidae) larvae. Rev Bras Parasitol Vet.

[52] Serpa MCA, Luz HR, Costa FB, Weck BC, Benatti HR, Martins TF (2021). Small mammals, ticks and rickettsiae in natural and human-modified landscapes: Diversity and occurrence of Brazilian spotted fever in Brazil. Ticks Tick Borne Dis.

[53] Randolph SE (2004). Tick ecology: processes and patterns behind the epidemiological risk posed by ixodid ticks as vectors. Parasitology.

[54] Oliveira PR. *Amblyomma cajennense* (Fabricius, 1787) (Acari: Ixodidae): Avaliação de técnicas para o estudo de dinâmica populacional e biotecnologia. Doctoral thesis. Belo Horizonte: Universidade Federal de Minas Gerais;1998 (in Portuguese).

[55] Labruna MB (2018). Comparative survival of the engorged stages of *Amblyomma cajennense* sensu stricto and *Amblyomma sculptum* under different laboratory conditions. Ticks Tick Borne Dis.

[56] Luz HR, Ramírez-Hernández A, Benatti HR, Ramirez DG, Szabó MPJ, Labruna MB (2020). Comparative survival of the engorged stages of *Amblyomma dubitatum* and *Amblyomma sculptum* in the laboratory: Implications for Brazilian spotted fever epidemiology. Ticks Tick Borne Dis.

[57] Queirogas VL, Del Claro K, Nascimento ART, Szabó MPJ (2012). Capybaras and ticks in the urban areas of Uberlândia, Minas Gerais, Brazil: ecological aspects for the epidemiology of tick-borne diseases. Exp Appl Acarol.

[58] Estrada-Peña A, Tarragona EL, Vesco U, Meneghi D, Mastropaolo M, Mangold AJ (2014). Divergent environmental preferences and areas of sympatry of tick species in the *Amblyomma cajennense* complex (Ixodidae). Int J Parasitol.

[59] Clifford CM, Anastos G, Van der Borght-Elbl A. The larval ixodid ticks of the eastern United States (Acarina-Ixodidae). Misc Publ Ann Entomol Soc Am. 1961;2:215–44.

[60] Martins TF, Onofrio VC, Barros-Battesti DM, Labruna MB (2010). Nymphs of the genus *Amblyomma* (Acari: Ixodidae) of Brazil: descriptions, redescriptions, and identification key. Ticks Tick Borne Dis.

[61] Dantas-Torres F, Fernandes Martins T, Muñoz-Leal S, Onofrio VC, Barros-Battesti DM. Ticks (Ixodida: Argasidae, Ixodidae) of Brazil: Updated species checklist and taxonomic keys. Ticks Tick Borne Dis. 2019;10:101252. 10.1016/j.ttbdis.2019.06.012 31255534

[62] Onofrio VC, Labruna MB, Pinter A, Giacomin FG, Barros-battesti DM. Comentários e chaves para as espécies do gênero *Amblyomma*. In: Barros-Battesti DM, Arzua M, Bechara GH, editors. Carrapatos de importância médico-veterinária da Região Neotropical: um guia ilustrado para identificação de espécies. São Paulo/Butantan: Vox/ICTTD-3; 2006. p. 53–114.

[63] Belozerov VN. Diapause and biological rhythms in ticks. In: Obenchain FD, Galun R, editors. Physiology of ticks. Oxford: Pergamon Press; 1982; p. 469–500.

[64] Cabrera RR, Labruna MB (2009). Influence of photoperiod and temperature on the larval behavioral diapause of *Amblyomma cajennense* (Acari: Ixodidae). J Med Entomol.

[65] Labruna MB, Amaku M, Metzner JA, Pinter A, Ferreira F (2003). Larval behavioral diapause regulates life cycle of *Amblyomma cajennense* (Acari: Ixodidae) in Southeast Brazil. J Med Entomol.

[66] Katz G, Neves VLFC, Angerami RN, Nascimento EMM, Colombo S (2009). Situação epidemiológica e importância da febre maculosa no Estado de São Paulo. BEPA.

[67] Szabó MPJ, Martins TF, Barbieri ARM, Costa FB, Soares HS, Tolesano-Pascoli GV (2020). Ticks biting humans in the Brazilian savannah: Attachment sites and exposure risk in relation to species, life stage and season. Ticks Tick borne Dis.

[68] Beck DL, Zavala J, Montalvo EO, Quintana FG (2011). Meteorological indicators for *Amblyomma cajennense* and population dynamics in the Tamaulipan Biotic Province in Texas. J Vector Ecol.

