# Supplementary material for: Seasonal dynamics of Amblyomma sculptum: a review
Source: Parasit Vectors. 2022 Jun 6;15:193. doi: 10.1186/s13071-022-05311-w (PMC9169286; doi:10.1186/s13071-022-05311-w)
Supplement: Supplementary file 1 — Additional file 1: Table S1. Additional data on collection methodology applied by Amblyomma sculptum seasonal dynamic studies between 1990 and 2022. [file 13071_2022_5311_MOESM1_ESM.docx]

Additional file 1: Table S1 Additional data on collection methodology applied by *Amblyomma sculptum* seasonal dynamic studies between 1990 and 2022.

| Dragging and Flagging | | | | CO_2_ Traps | | | | Visual Search | | Reference |
| --- | --- | --- | --- | --- | --- | --- | --- | --- | --- | --- |
| Duration (min) | Distance  (m) | Cloth size (m) | Pause for Inspection | Duration of exposure (min) | Cloth size | Number of traps | Dry ice/trap (g) | Duration (min) | Distance (m) |  |
| 200 | 80 | 1.25x0.8 | NI | - | - | - | - | - | - | Souza [43] |
| - |  | 1.8x0.7 |  |  | - | - | - | - | - | Lemos et al. [31] |
| 120 | 20 | 1.5x0.9 | NI | 90 | 1m^2^ | 4 | 500 | - | - | Oliveira et al. [32] |
| - | - | - | - | - | - | - | - | - | - | Labruna et al. [28] |
| - |  | - | - | - | - | - | - | - | - | Oliveira et al. [29] |
| - | - | - | - | 60 | 1m^2^ | 3 | 800 | - | - | de Souza et al. [16] |
| 90 | NI | NI | NI | 90 | NI | 6-14 per location | NI | - | - | Szabó et al. [14] |
| NI | 10 to 20 | 1.5x0.9 | NI | 120 | 1m^2^ | 4 | 500 | - | - | Toledo et al. [38] |
| 120 | NI | NI | NI | NI | NI | 5 | NI | - | - | Guedes and Cerqueira Leite [34] |
| 40 | NI | 1.5x0.9 | Every 20m | 40 | NI | 35 | NI | NI | NI | Veronez et al. [15] |
| - | - | - | - | NI | NI | 10-13 per location | NI | - | - | Brites-Neto et al. [46] |
| 60 | NI | 1.5x1 | Every 10m | - | - | - | - | - | - | Tarragona et al. [7] |
| 60 | NI | 2x1 | NI | 60 | 2x1m | 20 | NI | NI | 400 | Szabó et al. [39] |
| 90 | NI | NI | NI | NI | NI | 30 | NI | - | - | Barbieri et al. [35] |
| - | - | - | - | 120 | 1m^2^ | 19-20 | 500 | - | - | Dantas-Torres et al. [36] |
| 180 | NI | 1x0.75 | NI | - | - | - | - | 180 | NI | de Paula et al. [37] |
| 60 | 500 | NI | NI | NI | NI | 10 per location | NI | 60 | 500 | Garcia et al. [40] |

NI: not informed
