# Supplementary material for: Seasonal dynamics of Amblyomma sculptum: a review
Source: Parasit Vectors. 2022 Jun 6;15:193. doi: 10.1186/s13071-022-05311-w (PMC9169286; doi:10.1186/s13071-022-05311-w)
Supplement: Supplementary file 2 — Additional file 2: Table S2. Results from Amblyomma sculptum seasonal dynamic studies between 1990 and 2022 and mean photoperiod (PP: daylight length in hours) at the time of highest availability of each active stage. [file 13071_2022_5311_MOESM2_ESM.docx]

Additional file 2: Table S2 Results from *Amblyomma sculptum* seasonal dynamic studies between 1990 and 2022 and mean photoperiod (PP: daylight length in hours) at the time of highest availability of each active stage.

|  | Results | | |  |
| --- | --- | --- | --- | --- |
| Region | *Amblyomma* spp. Larvae | *Amblyomma* spp. Nymphs | *A. sculptum* Adults | Reference |
| Southeastern Brazil | Between June and September; PP: 11.4 | Between July and October; PP: 11.8 | Between February and April; PP: 12.2 | Souza [43] |
| Southeastern Brazil | Between April and September; PP:11.3 | Between June and November; PP: 11.8 | Between October and May; PP: 12.8 | Lemos et al. [31] |
| Southeastern Brazil | Between April and October; PP: 11.6 | Between May and September; PP: 11.4 | Between August and May; PP: 12.3 | Oliveira et al. [32] |
| Southeastern Brazil | Between April and June; PP: 11.3 | Between July and October; PP: 11.8 | Between October and March; PP: 12.9 | Labruna et al. [28] |
| Southeastern Brazil | Between April and August; PP: 11.3 | Between June and October; PP: 11.6 | Between September and March; PP: 12.8 | Oliveira et al. [29] |
| Southeastern Brazil | All year; PP: 12.1 | Between July and December; PP: 12.3 | Between October and April; PP: 12.8 | de Souza et al. [16] |
| Southeastern Brazil | Between Autumn and Winter; PP: 11.5 | Winter; PP: 11.3 | Between Summer and Spring; PP: 12.8 | Szabó et al. [14] |
| Southern Brazil | All year; PP: 12.1 | All year; PP: 12.1 | Between Spring and Summer (October and November); PP: 12.9 | Toledo et al. [38] |
| Southeastern Brazil | Between April and November; PP: 11.7 | Between June and November; PP: 11.8 | Between August and April; PP: 12.5 | Guedes and Cerqueira Leite [34] |
| Southeastern Brazil | Between Autumn and Winter; PP: 11.5 | Winter; PP: 11.2 | Spring; PP: 13.0 | Veronez et al. [15] |
| Southeastern Brazil | Between April and July; PP: 11.1 | Between June and October; PP: 11.9 | Between October and December; PP: 13.1 | Brites-Neto et al. [46]* |
| Northwestern Argentina | Beginning of Winter; PP: 10.8 | Mid spring; PP: 13.0 | Mid to end of Summer; PP: 12.6 | Tarragona et al. [7]* |
| Southeastern Brazil | Autumn; PP: 11.5 | Winter; PP:11.2 | Summer; PP: 12.3 | Szabó et al. [39]* |
| Southeastern Brazil | Autumn; PP: 11.7 | Between Winter and Spring; PP: 12.1 | Between Spring and Summer; PP: 12.6 | Barbieri et al. [35]* |
| Northeastern Brazil | Between February and august; PP: 12.0 | All year with peak during Spring; PP: 12.4 | All year with peak during Spring; PP: 12.4 | Dantas-Torres et al. [36]* |
| Midwestern Brazil | Between May and October; PP: 11.7 | Between June and November; PP: 11.9 | Between September and March; PP: 12.6 | de Paula et al. [37]* |
| Midwestern Brazil | Autumn (May) and Winter (August);  PP: 11.4 | Winter (August); PP: 11.5 | Spring (December); PP: 13.3 | Garcia et al. [40]* |

*Studies in which nymphs were identified to species level.
